# Injury to the thorax is a dominant contributor to post-traumatic neutrophil mediated inflammatory response

**DOI:** 10.1007/s00068-026-03221-5

**Published:** 2026-06-02

**Authors:** F. J. C. van Eerten, L. V. Duebel, E. J. de Fraiture, M. F. Hanlo, E. A. Breuking, S. R. Brands, T. D. van den Bosch, K. J. P. van Wessem, N. Vrisekoop, L. Koenderman, F. Hietbrink

**Affiliations:** 1 Department of Trauma Surgery, University Medical Center Utrecht, Utrecht, 3508 GA Netherlands; 2 Department of Respiratory Medicine and Center for Translational Immunology (CTI), University Medical Center Utrecht, Utrecht, The Netherlands

**Keywords:** Inflammation, Injury, Neutrophil, Trauma, Thorax

## Abstract

**Purpose:**

Traumatic injury induces a neutrophil associated inflammatory response mediated by the release of damage-associated molecular patterns (DAMPs). This response can be maladaptive, increasing risk of infection. Since blunt thoracic injuries are associated with high infection rates, we hypothesized that these injuries elicit a stronger neutrophil associated inflammatory response than injuries to other body regions.

**Methods:**

A cohort study was conducted in adult patients presenting at the trauma bay of a level 1 trauma center between March 2020 and November 2022. These patients underwent routine analysis of their neutrophil associated inflammatory response by phenotyping blood neutrophils using automated flow cytometry. Injury per body region was counted when the Abbreviated Injury Severity (AIS) scores were ≥3 to focus on substantial injuries. The primary outcome was an extensive post-traumatic inflammatory neutrophil response.

**Results:**

861 patients were included. Blunt internal thoracic injuries showed the strongest association with severe neutrophil associated inflammation (OR 5.7), followed by abdominal (OR 2.6), pelvis (OR 2.5) and lower extremities (OR 2.3), even when corrected for ISS, age and hemodynamic instability. In multivariable analysis, only thoracic internal injuries remained a statistically significant contributor to excessive neutrophil associated inflammation (OR 5.4).

**Conclusion:**

Injuries to internal thoracic organs elicit a more pronounced post-traumatic neutrophil associated inflammatory response than injuries of similar severity in other body regions. This might be due to greater release of DAMPs from the soft tissue of the lungs. Understanding the body region-specific activation of neutrophil associated inflammation may support more patient-tailored surgical strategies to lower the infection risk.

**Supplementary Information:**

The online version contains supplementary material available at 10.1007/s00068-026-03221-5.

## Introduction

Trauma constitutes 8% of total mortality worldwide [1]. With the survival rates of trauma improving, infectious complications are emerging as a substantial challenge in the care of severely injured patients. Currently, 30–50% of polytrauma patients develop infectious complications [2, 3], which significantly contributes to prolonged hospitalization, increased healthcare costs and secondary mortality rates after trauma [2]. A maladaptive post-traumatic neutrophil associated inflammatory response is believed to increase susceptibility to these infectious complications [4–6].

The cellular inflammatory response following trauma is a cascade of events initiated by damage-associated molecular patterns (DAMPs), released from damaged cells during tissue injury [4, 6]. Neutrophils are the most abundant innate immune effector cells in the peripheral blood and play a major role in this response [4, 6]. Recent evidence suggests that the functionality of these cells, rather than their cell count, is important in the predisposition of infectious complications in trauma patients [5, 7].

Previous research on neutrophil phenotypes showed that during severe acute inflammation, these cells can be categorized into distinct subsets based on specific surface markers, providing improved insight into the pathogenesis of inflammatory response compared to standard laboratory methods [5, 8]. Neutrophil phenotypes can be distinguished by surface expression of FcgRIII/CD16 and L-selectin/CD62L, resulting in neutrophil phenotype categories [5]. This is illustrated by the finding that inflammation is associated with the early mobilization from the bone marrow of CD16^low^ young neutrophils (“left-shift”) that is followed by the occurrence (around day 5–8 after trauma) of CD62L^dim^ neutrophils, i.e. hypersegmented neutrophils [7]. These latter neutrophils exhibit impaired killing of micro-organisms [7]. As such, it is suggested that patients with high numbers of these refractory cells show a higher risk of mortality or infectious complications [2].

The relationship between overall injury severity, reflected by the extent of tissue damage, and the magnitude of post-traumatic neutrophil associated inflammatory response has been established [2]. Thoracic trauma is frequently encountered in severely injured trauma patients and is associated with a high incidence of infectious complications, such as pneumonia [9] This suggests that thoracic injuries may play a role in shaping the post-traumatic neutrophil associated inflammatory response.

Several studies have examined the impact of thoracic trauma on this response, although results have been inconsistent [10, 11]. Penetrating chest injuries resulting in uncomplicated pneumo- or hematothorax have been reported to induce only a short and mild systemic inflammatory activation [11]. In contrast, another study described a pronounced inflammatory response following blunt thoracic trauma which was not compared the response to injury to other body regions [10].

Therefore, it was hypothesized that blunt thoracic trauma elicits a more pronounced neutrophil associated inflammatory response than injury to other body regions. A better understanding of the effect of blunt thorax trauma on the neutrophil associated inflammatory response compared to injuries to other body regions may provide clinical insight. This could support more personalized decision-making in trauma care as an addition to existing physiology-based approaches. In-depth knowledge on neutrophil inflammatory responses can help tailor the surgical load to the patient’s immunological state. Therefore, the aim of this study was to investigate the specific impact of blunt thoracic trauma to the post-traumatic inflammatory neutrophil associated response.

## Methods

### Study design

A single-center observational cohort study based on a registry with prospectively and freshly collected immune data, was conducted in the UMC Utrecht between 2020 and 2022. This study was approved by the UMCU ethical review committee (reg. no. 24U-0097_QoTc). The processing and storage of data were in accordance with privacy and ethics regulations. Patient data were retrospectively collected from the regional chapter of the Dutch National Trauma Registry (DNTR) [12]. Data on neutrophil phenotypes were collected using fully automated flow cytometry, as elaborated on below.

### Patient selection

Patients were selected if they were included in the DNTR and had undergone analysis of neutrophil phenotypes by fully automated flow cytometry. The DNTR includes all injured patients who present at the Emergency Department (ED) immediately (< 48 h) after trauma. It included all patients who were subsequently admitted to hospital, transferred to another hospital, or die during ED treatment or within 48 h post-trauma [12]. All patients were treated according to local implemented the Advanced Trauma Life Support algorithm [13]. Only patients *≥* 16 years old were included. There were no exclusion criteria on comorbidities such as immune disorders, alcohol and drugs abuse, as it has not been established that these comorbidities predispose for different neutrophil subset outcomes [5].

### Data collection

For each patient, age, gender, hemodynamic instability, Abbreviated Injury Scales (AIS), Injury Severity Scores (ISS) [14] and neutrophil phenotype category were collected [5]. In addition, outcomes on mortality and total length of hospital stay were extracted. Injury data were collected at presentation and were coded using the AIS system by dedicated fulltime data managers of the regional chapter of the DNTR [12]. AIS was based on clinical findings and radiological imaging (CT scans in combination with X-rays). Only injuries with an AIS *≥* 3 were scored as injury to the corresponding body region in order to include only substantial injuries [15–17]. Hemodynamic instability was defined as a systolic blood pressure < 90 mmHg at ED presentation.

### Sample collection and neutrophil phenotype categorization

Upon arrival at the trauma bay, a 4 mL sodium-heparin blood sample (Greiner Bio-One GmbH, Kremsmünster, Austria) was collected per patient for granulocyte phenotyping. Samples were immediately analyzed using the AQUIOS CL^®^ “load & go” flow cytometer (Beckman Coulter Life Sciences, USA). Flow cytometry examines the fluorescence of antibody/fluorochrome-combinations specific for different target proteins on the surface of immune cells in the blood, following the protocol by de Fraiture et al. [18].

**Fig. 1:**
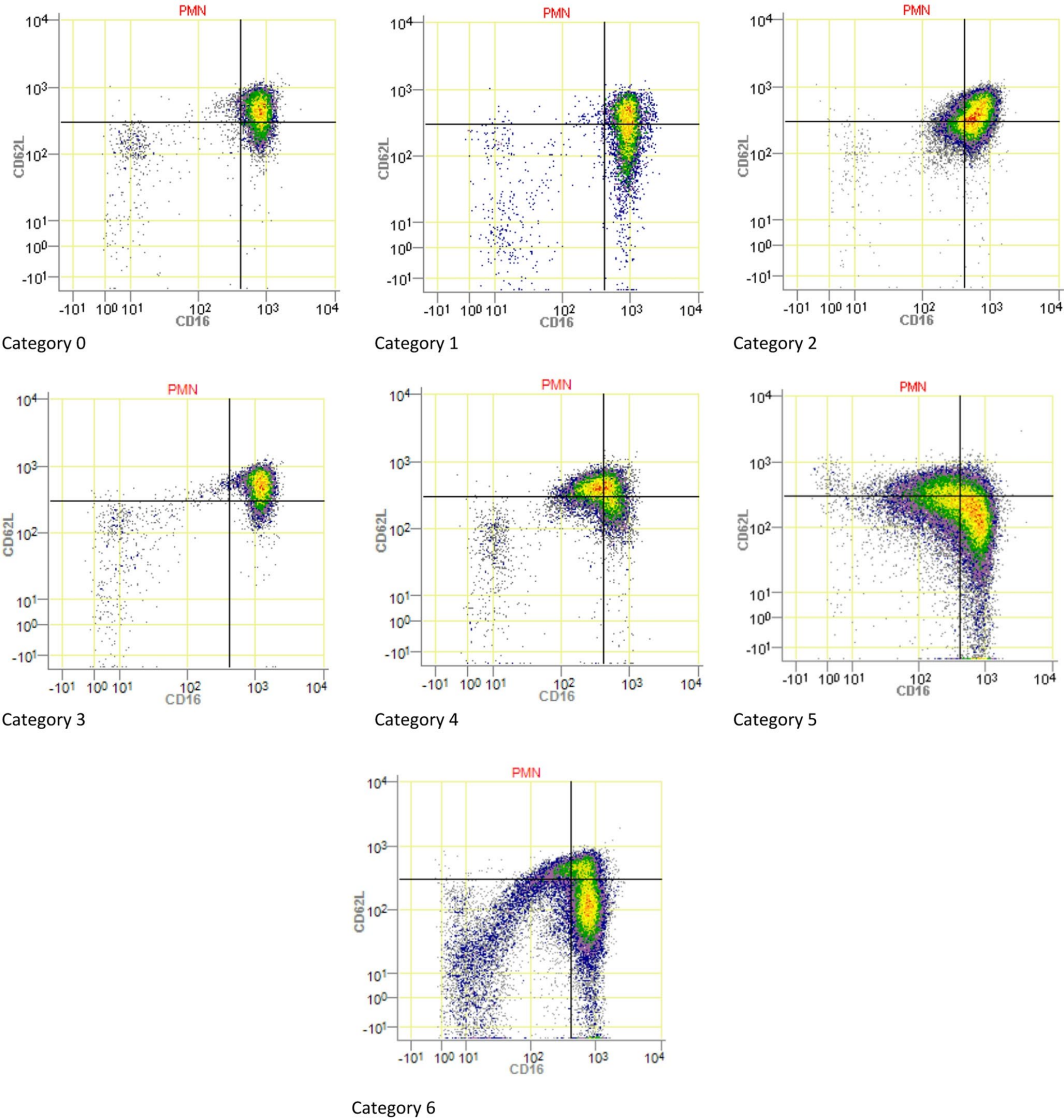
Examples of neutrophil phenotype categories, based on dot plots of granulocytes. **X-axis** expression of CD16/FcyRlll surface markers. **Y-axis** expression of CD62L/L-selectin neutrophil surface markers

Neutrophils were categorized based on their expression of CD16 and CD62L on their cell surface. The categories were determined as described in Fig. 1. A two-dimensional CD16/CD62L dot plot was visually categorized (0–6) based on these profiles, reflecting the composition of the different subsets, and the severity of the immune activation [5]. Categorization was performed through visual assessment by two independent and blinded researchers; discrepancies were resolved by a third observer.

**Fig. 2:**
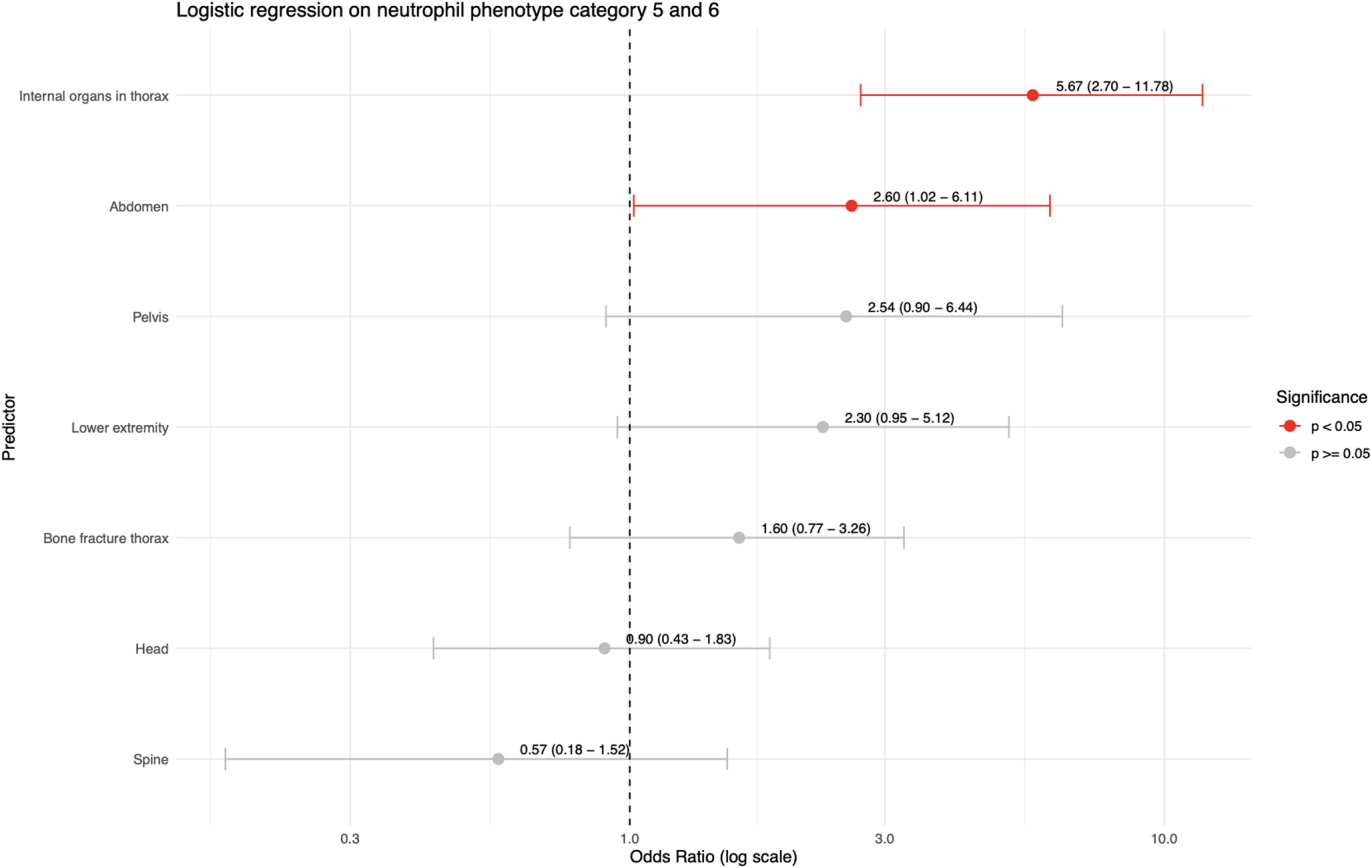
Logistic univariable regression on outcome neutrophil phenotype category 5 or 6; i.e. extensive post-traumatic neutrophil inflammatory response. Each predictor was analyzed separately, adjusting for Injury Severity Score (ISS), age and hemodynamic instability as confounders. Hemodynamic instability was defined as a systolic blood pressure < 90 mmHg at emergency department arrival

**Fig. 3:**
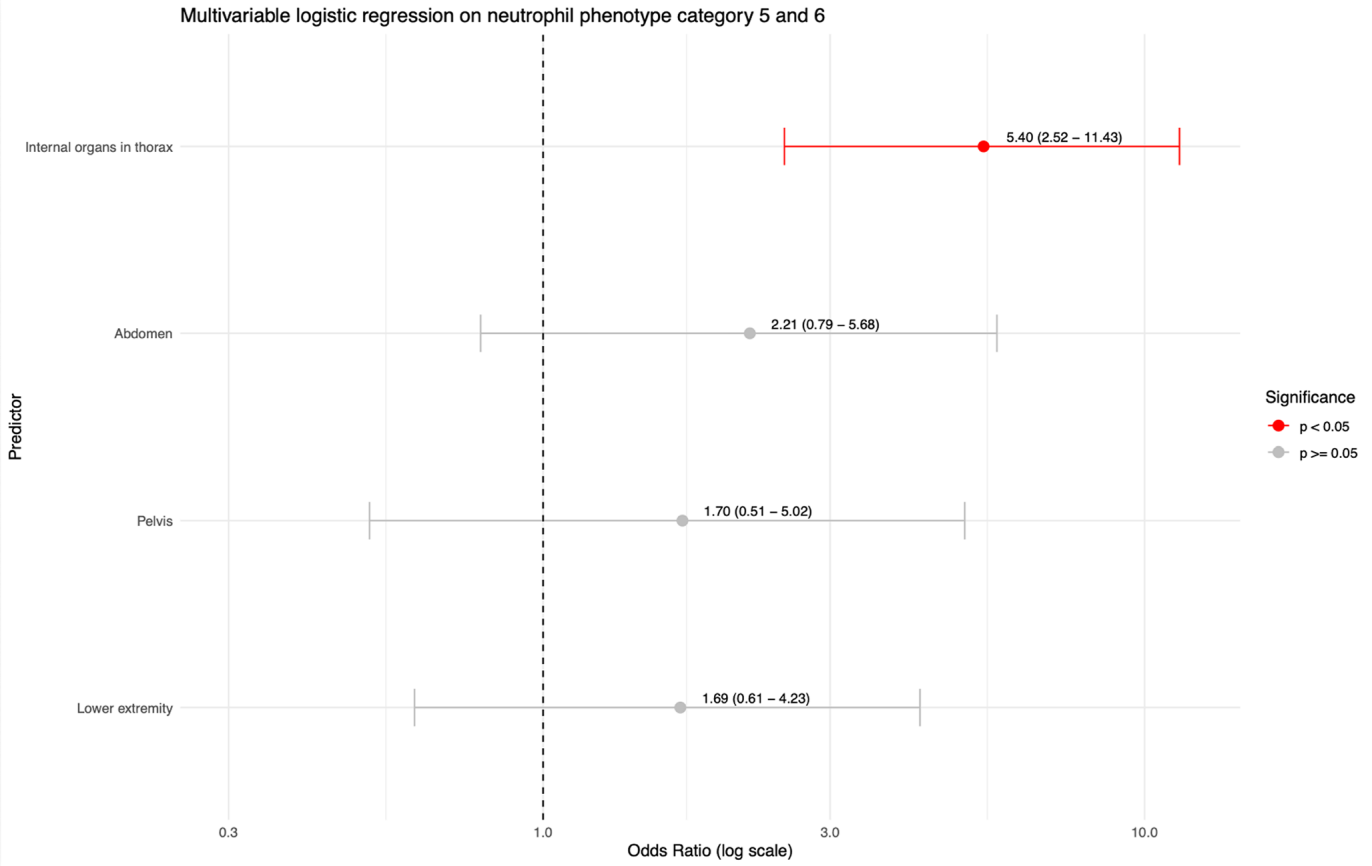
Logistic multivariable regression on outcome neutrophil phenotype category 5 or 6; i.e. extensive post-traumatic neutrophil inflammatory response. The predictors were analyzed together in one model, adjusting for Injury Severity Score (ISS), age and hemodynamic instability as confounders. Hemodynamic instability was defined as a systolic blood pressure < 90 mmHg at emergency department arrival

**Fig. 4:**
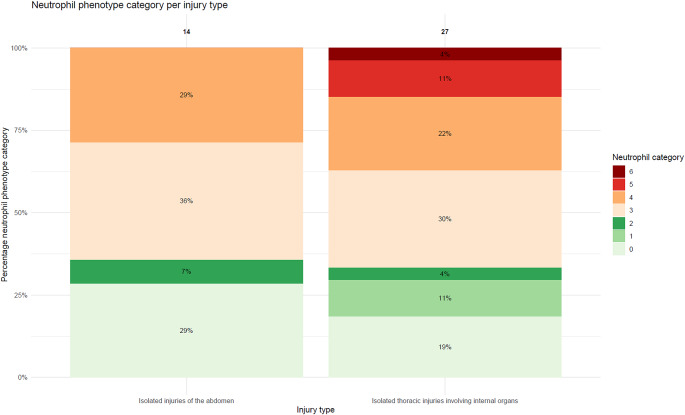
Distribution of neutrophil phenotypes per patients with injuries to isolated abdomen (*n* = 14) and patients with isolated thoracic injuries involving internal organs (*n* = 27) **X-axis** injury type. **Y-axis** percentage within each neutrophil phenotype category

**Fig. 5:**
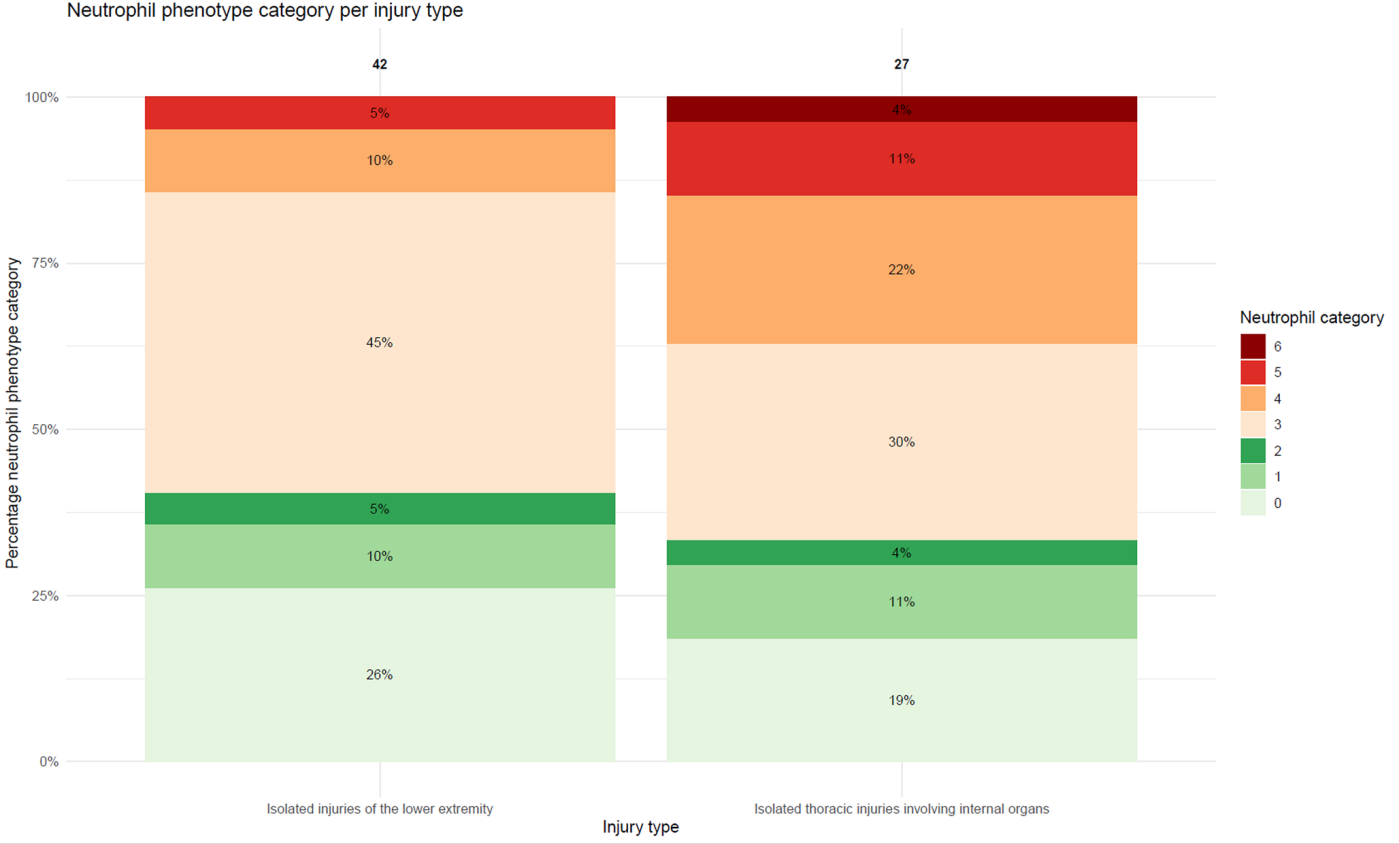
Distribution of neutrophil phenotypes per patients with isolated injuries to the lower extremities (*n* = 42) and patients isolated thoracic injuries involving internal organs (*n* = 27) **X-axis** injury type. **Y-axis** percentage within each neutrophil phenotype category

**Table 1:** Baseline and outcome characteristics of included patients

	Overall		Neutrophil phenotype category																
			0		1		2		3		4		5		6				
	***n/median***	***(%) / [IQR]***	***n/median***	***(%) / [IQR]***	***n/median***	***(%) / [IQR]***	***n/median***	***(%) / [IQR]***	***n/median***	***(%) / [IQR]***	***n/median***	***(%) / [IQR]***	***n/median***	***(%) / [IQR]***	***n/median***	***(%) / [IQR]***	***p-value***		
Number of patients	861		203	(23.6)	81	(9.4)	23	(2.7)	324	(37.6)	185	(21.5)	35	(4.1)	10	(1.2)			
Age	53	[32–70]	44	[28–67]	48	[30–67]	28	[21–40]	56	[38–70]	58	[37–73]	49	[26–69]	46	[32–53]	< 0.001		
Sex (F)	248	(28.8)	62	(30.5)	20	(24.7)	4	(17.4)	91	(28.1)	60	(32.4)	8	(22.9)	3	(30.0)	0.64		
Glasgow coma scale < 8	156	(18.2)	26	(12.9)	14	(17.5)	2	(8.7)	50	(15.5)	44	(23.9)	11	(30.6)	9	(90.0)	< 0.001		
Injury severity score	13	[9–21]	10	[5–14]	10	[5–17]	12	[9–14]	13	[9–18]	17	[10–25]	26.0	[17–34]	35	[30–44]	< 0.001		
Hemodynamic unstable	31	(3.6)	1	(0.5)	1	(1.2)	0	(0.0)	9	(2.8)	10	(5.4)	6	(17.1)	4	(40.0)	< 0.001		
%Banded neutrophils	5.0	[2.3–13.7]	1.0	[0.6–3.0]	3.9	[1.8–7.7]	12.7	[6.3–20.6]	3.4	[2.3–7.1]	13.2	[7.5–22.1]	27.3	[22.1–36.9]	22.5	[15.8–28.4]	< 0.001		
Injury per region (AIS *≥* 3)																			
Head	273	(31.7)	56	(27.6)	25	(30.9)	6	(26.1)	100	(30.9)	64	(34.6)	18	(51.4)	4	(40.0)	0.15		
Face	15	(1.7)	2	(1.0)	1	(1.2)	0	(0.0)	5	(1.5)	6	(3.2)	1	(2.9)	0	(0.0)	0.66		
Neck	12	(1.4)	1	(0.5)	1	(1.2)	0	(0.0)	3	(0.9)	7	(3.8)	0	(0.0)	0	(0.0)	0.11		
Thorax	261	(30.3)	33	(16.3)	18	(22.2)	6	(26.1)	94	(29.0)	82	(44.3)	21	(60.0)	7	(70.0)	< 0.001		
Thorax bone fracture	221	(25.7)	26	(12.8)	13	(16.0)	6	(26.1)	83	(25.6)	73	(39.5)	15	(42.9)	5	(50.0)	< 0.001		
Thorax internal organs	83	(9.6)	7	(3.4)	5	(6.2)	2	(8.7)	24	(7.4)	24	(13.0)	16	(45.7)	5	(50.0)	< 0.001		
Abdomen*	46	(5.3)	4	(2.0)	0	(0.0)	2	(8.7)	16	(4.9)	14	(7.6)	9	(25.7)	1	(10.0)	< 0.001		
Hollow organs	11	(1.3)	1	(0.5)	0	(0.0)	0	(0.0)	4	(1.2)	4	(2.2)	2	(5.7)	0	(0.0)	0.17		
Solid organs	31	(3.6)	3	(1.5)	0	(0.0)	1	(4.3)	10	(3.1)	10	(5.4)	7	(20.0)	0	(0.0)	< 0.001		
Spine	87	(10.1)	5	(2.5)	6	(7.4)	3	(13.0)	37	(11.4)	29	(15.7)	5	(14.3)	2	(20.0)	0.001		
Upper extremities**	15	(1.7)	4	(2.0)	2	(2.5)	0	(0.0)	7	(2.2)	2	(1.1)	0	(0.0)	0	(0.0)	0.89		
Soft tissue injury	2	(0.2)	2	(1.0)	0	(0.0)	0	(0.0)	0	(0.0)	0	(0.0)	0	(0.0)	0	(0.0)	0.37		
Bone fracture	13	(1.5)	2	(1.0)	2	(2.5)	0	(0.0)	7	(2.2)	2	(1.1)	0	(0.0)	0	(0.0)	0.80		
Lower extremities**	75	(8.7)	16	(7.9)	4	(4.9)	3	(13.0)	24	(7.4)	18	(9.7)	10	(28.6)	0	(0.0)	0.002		
Soft tissue injury	7	(0.8)	3	(1.5)	0	(0.0)	0	(0.0)	2	(0.6)	2	(1.1)	0	(0.0)	0	(0.0)	0.85		
Bone fracture	66	(7.7)	13	(6.4)	4	(4.9)	3	(13.0)	22	(6.8)	15	(8.1)	9	(25.7)	0	(0.0)	0.003		
Pelvis	37	(4.3)	0	(0.0)	0	(0.0)	1	(4.3)	12	(3.7)	16	(8.6)	7	(20.0)	1	(10.0)	< 0.001		
Total length of stay***	5	[3–12]	3	[2–6]	4	[2–14]	4	[2–9]	5	[3–11]	8	[4–14]	13	[6–21]	3	[2–7]	< 0.001		
ICU length of stay***	3	[2–7]	2	[1–4]	4	[2–6]	4	[2–5]	3	[2–5]	4	[2–7]	7	[4–10]	3	[2–5]	0.008		
30 days mortality	78	(9.1)	6	(3.0)	4	(4.9)	1	(4.3)	27	(8.3)	28	(15.1)	4	(11.4)	8	(80.0)	< 0.001		

*The abdomen region included also whole area and vessel injuries next to hollow- and solid organ injury. **The upper and lower extremity regions also included vessel and nerve injuries next to soft tissue injury and bone fracture. Lower extremities did not include pelvic injury. ***The length of stay was calculated only for patients admitted to the hospital and **ICU** (Intensive Care Unit), respectively. **AIS** Abbreviated Injury Scale. Hemodynamic instability was defined as a systolic blood pressure < 90 mmHg at emergency department arrival

**Table 2:** Distribution of neutrophil phenotype category per abdominal and thorax internal organ and bone injuries

	Abdominal injury without thorax internal organ injury		Thorax internal organ injury without abdominal injury		
	*n*/median	(%)/[IQR]	*n*/median	(%)/[IQR]	*p*-value
Total number of patients	14		27		
Age	36	[28–42]	43	[28–57]	0.56
Sex (F)	2	(14,3)	4	(14,8)	1.00
Median AIS of associated body region	3	[3–3]	3	[3–3]	0.87
Injury Severity Score	14	[12–17]	13	[10–17]	0.72
* ≥* 2 body regions AIS *≥* 3	0	(0,0)	0	(0,0)	
Injury to region with AIS *≥* 3					
Head	0	(0,0)	0	(0,0)	
Thorax	0	(0,0)	27	(100,0)	
Abdomen	14	(100,0)	0	(0,0)	
Spine	0	(0,0)	0	(0,0)	
Upper extremity	0	(0,0)	0	(0,0)	
Lower extremity	0	(0,0)	0	(0,0)	
Pelvis	0	(0,0)	0	(0,0)	
Neutrophil phenotype category					0.48
0	4	(28,6)	5	(18,5)	
1	0	(0,0)	3	(11,1)	
2	1	(7,1)	1	(3,7)	
3	5	(35,7)	8	(29,6)	
4	4	(28,6)	6	(22,2)	
5/6	0	(0,0)	4	(14,8)	
%Banded neutrophils	4.9	[2.8–12.6]	11.8	[4.3–24.3]	0.43

	Lower extremity injury without thorax internal organ injury		Thorax internal organ injury without lower extremity injury		
	n/median	(%)/[IQR]	n/median	(%)/[IQR]	p-value
Total number of patients	42		27		
Age	50	[29–64]	43	[28–57]	0.54
Sex (F)	11	(26,2)	4	(14,8)	0.41
Median AIS of associated body region	3	[3–3]	3	[3–3]	0.004
Injury Severity Score	10	[9–13]	13	[10–17]	0.005
* ≥* 2 body regions AIS *≥* 3	0	(0,0)	0	(0,0)	
Injury to region with AIS *≥* 3					
Head	0	(0,0)	0	(0,0)	
Thorax	0	(0,0)	27	(100,0)	
Abdomen	0	(0,0)	0	(0,0)	
Spine	0	(0,0)	0	(0,0)	
Upper extremity	0	(0,0)	0	(0,0)	
Lower extremity	42	(100,0)	0	(0,0)	
Pelvis	0	(0,0)	0	(0,0)	
Neutrophil phenotype category					0.39
0	11	(26,2)	5	(18,5)	
1	4	(9,5)	4	(14,8)	
2	2	(4,8)	1	(3,7)	
3	19	(45,2)	8	(29,6)	
4	4	(9,5)	6	(22,2)	
5/6	2	(4,8)	4	(14,8)	
%Banded neutrophils	3.0	[2.1–9.2]	11.8	[4.3–24.3]	0.045

Moreover, the proportion of banded neutrophils was determined using FlowSOM, an automated clustering algorithm that has been previously validated [5, 19]. This approach employs self-organizing maps that enable high-dimensional clustering and visualization of flow cytometry datasets. All markers from the flow cytometry panel (CD10, CD11b, CD16, CD62L, CD64) were included in the analyses. FlowSOM consistently delineated three neutrophil metaclusters: (1) mature neutrophils (CD16^bright^/CD62L^bright^), (2) banded neutrophils (CD16^dim^/CD62L^bright^), and (3) hypersegmented neutrophils (CD16^bright^/CD62L^dim^). The frequency of cells within each metacluster was quantified and subsequently exported.

### Statistical analysis

All analyses were performed using R for statistical computing (version 2024.12.0.46) [20]. Numerical variables were presented in median and interquartile range (IQR) and categorical variables were presented in numbers and percentages (%). Baseline characteristics were compared across the different neutrophil phenotype categories. Categorical variables were analyzed using the Chi-square test, and continuous variables were analyzed using the Kruskal-Wallis test. For variables showing significant differences between neutrophil categories, post-hoc analyses were performed to determine which categories differed from each other: pairwise comparisons of proportions for categorical variables and the Mann-Whitney-Wilcoxon test for continuous variables (both with Bonferroni correction) [21].

A logistic regression on severe post-traumatic neutrophil associated inflammatory response (neutrophil phenotype categories 5 and 6) was performed. Firstly, an univariable logistic regression was performed. The presence of injury to each body region was entered as a binary independent variable (yes/no), neutrophil phenotype categories 5 or 6 was the dependent variable. The regressions were was adjusted for age, ISS and hemodynamic instability. Adjustment for the ISS was performed to compare post-traumatic neutrophil associated inflammatory responses between body regions at comparable levels of overall injury severity. Only body regions that occurred in more than five patients were included. Results were expressed as odds ratios (OR) with 95% confidence intervals. A p-value < 0.05 was considered statistically significant and a non-significant trend was defined as a p-value < 0.20 [22]. Predictors that showed a statistically significant association or non-significant trend with severe post-traumatic neutrophil associated inflammatory response in the univariable regression were then included in a multivariable logistic regression model adjusting for each injured body region as well as overall ISS, age and hemodynamic instability [22].

Additionally, using a similar approach, both univariable and multivariable linear regression analyses were performed on the percentage of banded neutrophils, as determined by FlowSOM [19] (see above). These models were likewise adjusted for ISS, age and hemodynamic instability, and regression coefficients (β) with 95% confidence intervals were reported. A p-value < 0.05 was considered statistically significant and a p-value < 0.20 as non-significant trend.

To investigate differences in the extent of the neutrophil associated inflammatory response among patients with thoracic injuries, these patients were compared with individuals who sustained abdominal or lower extremity injuries, analyzed as two separate groups. The comparison with abdominal injuries was performed to evaluate variation in the response to soft-tissue trauma, whereas the comparison with lower extremity injuries (excluding pelvic fractures) was performed to assess differences between soft-tissue injuries and bony fractures. Patients with penetrating injuries or isolated thoracic bone fractures were excluded. Patients were subsequently divided into the following subgroups using the AIS codes [17, 23]. (See Table S1 for the AIS codes used for identification).

- Firstly, patients with isolated thoracic injuries involving internal organs AIS *≥* 3 were compared to patients with isolated abdominal injuries AIS *≥* 3 to determine regional variation in effect on the post-traumatic neutrophil associated inflammatory response.
- Secondly, patients with isolated thoracic injuries involving internal organs AIS *≥* 3 were compared to patients with isolated lower extremity injuries AIS *≥* 3 to establish the difference between soft-tissue and extremity injuries. Due to the low number (< 10) of patients with severe soft tissue injuries of the lower extremity (AIS *≥* 3), no distinction was made between bone and soft tissue injuries in this region.

Injuries were considered isolated when no additional body region had an AIS score *≥* 3. The distribution of neutrophil phenotype categories in the different body regions described above was compared using chi-square test.

## Results

### Patient characteristics

In total, 861 patients were included. The median age of all patients was 53 [IQR 32–70] and most of the patients were male: 613 (71.2%). The median ISS was 13 [IQR 9–21]. The most frequently injured body region was the head: 273 (31.7%) patients, which was followed by the thorax: 261 (30.3%) patients. The total length of hospital stay of all patients had a median of 5 [IQR 3–12] days, and the total median length of ICU stay was 3 [2–7] days. Overall mortality was 78 patients (9.1%) (Table 1).

### Patient characteristics and outcomes per neutrophil phenotype category

Of the 861 patients, most patients were classified as category 0 (203; 23.6%), category 3 (324; 37.6%) and category 4 (185; 21.5%) (see Table 1). The ISS increased progressively from categories 0–2 to 6; ranging from a median of 10 [IQR 5–14] in category 0 to 35 [IQR 30–44] in category 6 (Table 1). Post-hoc pairwise comparisons demonstrated significant differences, particularly between the lower (0–2) and higher (4–6) neutrophil phenotype categories (*p* < 0.001) (Table S2).

Length of hospital stay also increased across categories, from 3 days [IQR 2–6] in category 0 to 13 days [IQR 6–21] in category 5. Post-hoc tests showed significant differences (all *p* < 0.05) between categories 4 and 5 compared with categories 0–2 (Table S2).

The mortality rates increased progressively from neutrophil categories 0–2 to 6, remaining below 5% in categories 0–2 and rising from 8.3% in category 3 to 80% in category 6 (Table 1). Post-hoc pairwise comparison showed significant differences between category 6 to all other categories (*p* < 0.001) (Table S2).

### Univariate regression on neutrophil phenotype categories 5–6

In the univariate regression analysis on severe neutrophil associated inflammatory responses (i.e. neutrophil phenotype categories 5 or 6), blunt thoracic internal organ injuries had the highest odds ratio (OR 5.7, 95% CI 2.7–11.8; *p* < 0.001). Abdominal injuries (OR 2.6, 95% CI 1.0–6.1; *p* = 0.03) additionally showed significantly increased odds ratios. Pelvic injuries (OR 2.5, 95% CI 0.9–6.4; *p* = 0.06) and lower extremity injuries (OR 2.3, 95% CI 0.1–5.1; *p* = 0.05) showed non-significant trends of increased odds ratios to neutrophil associated inflammatory response. No significant findings were observed for thorax bone fractures, head and spine injuries (Fig. 2).

### Multivariable regression on neutrophil phenotype categories 5–6

Injuries to the internal organs of the thorax, abdomen, pelvis and lower extremities were included in the multivariable regression on the occurrence of a severe neutrophil associated inflammatory response. The multivariable regressions were corrected for age, ISS, hemodynamic instability and the other injured body regions. Of these predictors, only blunt injuries to internal organs of the thorax yielded a significant association (OR 5.4, 95% CI 2.5–11.4; *p* < 0.001). No significant associations were found for injuries to the abdomen (OR 2.2, 95% CI 0.8–5.7; *p* = 0.11), pelvis (OR 1.7, 95% CI 0.5–5.0; *p* = 0.36) and lower extremity injuries (OR 1.7, 95% CI 0.6–4.2; *p* = 0.28) (Fig. 3).

### Linear regression on percentage of banded neutrophils

The univariate linear regression on percentage of banded neutrophils showed significant ß-coefficient for thoracic internal organ injuries and thorax bony injuries: (ß 5.7, 95% CI 2.0–9.3; *p* = 0.002) and (ß 2.9, 95% CI 0.4–5.4; *p* = 0.02) respectively (Figure S1).

Injuries to the lower extremities showed a non-significant trend toward higher ß-coefficient of banded neutrophils (ß 3.3, 95% CI − 0.5–7.1, *p* = 0.08) (Figure S1).

When pooled in the multivariable linear regression, thorax internal organ injuries showed a significant ß-coefficient (ß 4.9, 95% CI 1.2–8.6; *p* = 0.01) on presence of banded neutrophils. The injuries to lower extremities and bone fractures of the thorax did not yield a significant ß-coefficient: (ß 3.2, 95% CI -0.5–6.7; *p* = 0.09 and ß 2.1, 95% CI -0.4–4.7; *p* = 0.10, respectively) (Figure S2).

### Thoracic and abdominal injuries

Among the patients, 14 patients suffered isolated abdominal injuries, and 27 patients sustained blunt isolated thoracic injuries involving internal organs AIS *≥* 3 (Table 2, Figure S3). Both thorax internal organs and abdominal AIS were a median of 3 [3–3]. The distribution of patients across neutrophil phenotype categories did not differ significantly between the two groups; extreme neutrophil associated inflammatory response occurred in 0 (0.0%) patients with abdominal trauma and in 4 (14.8%) thoracic (*p* = 0.48). Moreover, the median percentage of banded neutrophils did not differ significantly between the patient with abdominal and thoracic injuries; 4.9. [IQR 2.8–12.6] vs. 11.8 [IQR 4.3–24.3] percent, respectively (*p* = 0.41) (Fig. 4; Table 2).

### Thoracic and lower extremity injuries

In total, 42 patients suffered from isolated lower extremity AIS *≥* 3 (Table 2, Figure S3). Both thorax internal organs and lower extremity AIS were a median of 3 [3–3]. There was no significant difference in the number of patients with an extreme neutrophil associated inflammatory response in the thoracic injury group vs. lower extremity group; 4 (14.8%) vs. 2 (4.8%) patients (*p* = 0.39). Moreover, patients who suffered from injuries to the internal organs of the thorax had higher median percentage of banded neutrophils compared to patients who suffered from lower extremity injuries; 11.8 [IQR 4.3–24.3] vs. 3.0 [IQR 2.1–9.2] percent, respectively (*p* = 0.045) (Fig. 5; Table 2).

## Discussion

This study showed that, particularly thoracic injuries with damage to the internal thoracic organs were associated with an extensive post-traumatic neutrophil associated inflammatory response. This innate immune response was significantly less pronounced in patients who suffered abdominal, or severe lower extremity injuries. This difference was found even after correction for injuries to other body regions, ISS, age and hemodynamic instability. Regardless of injured body region, patients who suffered from an extensive neutrophil associated inflammatory response experienced longer length of hospital stay and higher mortality rates.

### Thoracic injuries

Multiple studies have demonstrated that thorax trauma is associated with an increased risk of complications compared to severely injured patients without thorax involvement [9, 24, 25]. Some of this elevated risk was thought to be attributable to situational factors, such as intubation [9]. This current study suggests that a severe immune response may also play a role in the increased risk of complications in thorax trauma patients. This finding is in line with previous research observing a more severe inflammatory response in animal models or patients with (femur) fractures combined with thoracic injuries vs. isolated (femur) fractures [26–31]. Moreover, prior research recognizes severe thoracic injuries as a key factor in surgical decision-making due to their elevated risk of complications [32, 33].

The occurrence of a more severe response of the neutrophil compartment in thorax injury patients might be explained by two mechanisms: Firstly, injuries to organs and soft tissue may be accompanied by more extensive release of DAMPs compared to bone fractures. Secondly, the narrow microvasculature of the lungs possibly cause stagnation of neutrophils in the lung capillaries.

### Soft tissue injuries

Understanding the differences in neutrophil associated inflammatory responses between injury types requires inside knowledge into the underlying pathophysiological mechanisms. It was previously demonstrated that damage-associated molecular patterns (DAMPs) and microbe derived pathogen-associated molecular patterns (PAMPs) contribute the neutrophil associated inflammatory response [34–38]. DAMPs are released from damaged host cells and include molecules such as ATP, HMGB1, cold-inducible RNA-binding protein, histones, and mitochondrial DNA and formyl peptides [37, 39]. PAMPs, on the other hand, originate from microbes and are typically introduced through direct injuries to hollow organs, such as intestinal injuries, or via open fractures [37]. The outcome of the current study suggests that soft-tissue injuries induce more DAMPs and/or PAMPs compared to isolated bony injuries. This may be explained by the higher cellular density in soft organ tissue compared to osseous tissue, which is primarily heavily mineralized extracellular matrix [40].

### Microvasculature of the lung compared with that of abdominal organs

Abdominal injuries may produce a similar number of DAMPs and PAMPs compared to the situation with soft-tissue thoracic injuries. Specifically, during intestinal injury, a high load of PAMPs may be released from the intestinal microbiome [37] and one might expect a comparable neutrophil associated inflammatory response compared to thorax trauma. However, this current study suggests that injuries to the thoracic internal organs causes a more extensive neutrophil associated inflammatory response compared to abdominal injury. One possible explanation is the unique anatomy of the pulmonary microvasculature, in which capillary diameters are smaller than the size of a neutrophil [41–44]. In addition, functional shunts to circumvent this are absent [41–44]. Following lung injury, many neutrophils are recruited to the alveolar vasculature where they become mechanically trapped [41]. This entrapment is exacerbated during inflammation, as (pre)activated neutrophils will increase in size and stiffness [42, 43]. As a result, microcirculatory flow is disturbed and the stay of (pre)activated cells in this compartment is prolonged [42–44]. These trapped neutrophils can then locally and systemically amplify inflammation, resulting in an increased overall innate inflammatory response [41–44].

### Extremity injury

Extremity injuries may induce a variable response of the neutrophil compartment depending largely on the extent of the associated soft tissue damage. For instance, a previous mouse study showed increased release of DAMPs in blast-injury after tourniquet induced ischemia to the extremities compared to no tourniquet usage [45]. These findings suggest that the soft-tissue damage associated with extremity injuries, rather than the fractures itself, largely drives the release of DAMPs. In our study, fractures of the extremities predominantly occurred in patients with neutrophil phenotype categories 0–3, suggesting only a moderate effect of these injuries on the development of a post-traumatic neutrophil associated inflammatory response. In contrast, univariate regression analysis showed that lower extremity fractures were associated with increased odds ratios of an extensive neutrophil associated inflammatory response.

Lower extremity fractures typically result from a high-impact trauma and are often accompanied by substantial collateral soft-tissue damage at other regions. This injury-severity association likely explains why the association lost significance in the multivariate analysis. This, however, remains speculation based on the hypothesis that soft-tissue injuries release more DAMPs than bony injuries [33, 34].

### Traumatic brain injury

Interestingly, traumatic brain injury was associated with neither an exacerbating nor protective effect of post-traumatic neutrophil associated inflammation. This aligns with our previous study that showed higher ISS in traumatic brain injury patients compared to patients with the same neutrophil categories without brain injuries [46]. This suggested that for traumatic brain injury, the correlation between overall tissue damage (DAMPs release) and neutrophil associated inflammatory response is limited [46].

### Biological background

Following traumatic injury, the release of DAMPs is thought to trigger the release of immature banded (CD16^low^/CD62L^high^) neutrophils from the bone marrow [8]. These banded cells exhibit enhanced bacterial containment/killing and increased phagolysosomal acidification capacity [47, 48]. In contrast, hypersegmented (CD16^high^/CD62L^low^) neutrophils are present in lower numbers immediately after trauma but increase significantly after 6 days post-injury [7]. Compared to banded neutrophils, hypersegmented cells have reduced anti-bacterial activity, and a lower capacity to acidify their phagolysosomes. On the other hand, they have the ability to suppress T cells by which they are involved in immune regulation [49]. Unlike banded neutrophils, the origin of hypersegmented neutrophils remains unclear, particularly after day 6 post-trauma [7]. However, they have been identified as a distinct neutrophil subset based on protein expression profiles and in vivo pulse-chase labeling [50].

### Clinical relevance

Previous studies have shown that a maladaptive post-traumatic neutrophil associated inflammatory response increases the risk of inflammatory and/or infectious complications [5]. It is speculated that an early excessive influx of banded neutrophils is associated with depletion of the neutrophil compartment in the bone marrow around day 5, which coincides with the observed peak in infection susceptibility between days 5 and 8 after trauma [5]. Moreover, a dysregulated immune response also reduces the patient’s ability to cope with a ‘second hit’, such as additional surgical interventions [51, 52]. These surgical procedures are considered to lead to extra tissue injury, which increases the risk of further depleting the neutrophil pool and potentially aggravating trauma-induced immunodeficiency [53, 54].

In recent years, surgical decision-making has become increasingly patient-tailored [55]. The choice for an early appropriate care approach, which includes damage control principles with multiple shorter interventions rather than one extensive procedure, is usually based on the patient’s physiological condition [52]. Several studies have investigated the integration of immunological status into this decision-making. However, no clinically useable and rapidly available marker has yet been identified [56]. Neutrophil phenotyping may provide such a tool. As fully automated flow cytometry has become available it can be performed near the bedside within a clinically relevant timeframe (< 30 min). It could support individualized surgical strategies by identifying patients at high risk for post-traumatic infectious and inflammatory complications [5]. Importantly, incorporating the inflammatory response into surgical decision-making may be beneficial as an adjunct to, rather than a replacement for, current parameters used to assess physiological instability.

Understanding the differential impact of various tissue injuries on the post-traumatic neutrophil associated inflammatory response could further improve insight into the role of the immune system in adverse outcomes. Future research should focus on elucidating the pathophysiological mechanisms underlying organ-specific characteristics leading to difference neutrophil responses.

### Limitations of the study

Several limitations should be mentioned. Firstly, the single-center design limits generalizability, as trauma patterns, including injury mechanisms and severity, may differ across settings. Secondly, sampling was restricted to admission only; longitudinal measurements would have enabled assessment of temporal neutrophil dynamics and their relation to clinical outcomes. Thirdly, residual confounding cannot be excluded. Although ISS and age were adjusted for, other relevant factors such as more in-depth data on severity of shock, and comorbidities were not incorporated, because this was not available in the data. In addition, statistical significance was not reached for several anatomical regions. Despite the large cohort, even greater statistical power would be needed to detect effects across all subgroups. Another limitation of this study is that neutrophil responses are time dependent and it is unknown whether the outcomes were influenced by differences in prehospital transportation time between patient groups. However, transportation times in the Netherlands are consistently under one hour and experimental data suggest stability of banded neutrophil counts within the first hours after trauma [57–60]. Therefore, any potential impact on our findings is likely minimal.

Lastly, this study did not investigate the association between neutrophil phenotype categories and clinical outcomes. To support the implementation of neutrophil phenotyping in surgical decision-making, future studies must investigate whether neutrophil phenotyping can be used for risk stratification.

## Conclusion

This study showed that traumatic injuries to the internal organs particularly of the thorax contribute to the escalation of the post-traumatic neutrophil associated inflammatory response. Applying data underlying the effect of different injuries on the reaction of the neutrophil compartment should be incorporated into the decision-making on surgical strategies, because this may reflect the physiological reserve to tolerate surgical interventions. Assessment of the early neutrophil associated response may provide additional insight into a patient’s physiological state and offers a readily available tool to personalize trauma care, with the goal of preventing infectious complications.

## Supplementary Information

Below is the link to the electronic supplementary material.

Supplementary Material 1

## Data Availability

Restrictions apply to the availability of the data supporting the findings of this study, as they were used under license for the current study and are not publicly available. However, the data can be obtained from the authors upon reasonable request and with permission from University Medical Center Utrecht.

## References

[1] World Health Organization. Injuries and violence [Internet]. 2024 [cited 2025 May 1]. https://www.who.int/news-room/fact-sheets/detail/injuries-and-violence#. Accessed 1 May 2025.

[2] de Fraiture EJ, Vrisekoop N, Leenen LPH, van Wessem KJP, Koenderman L, Hietbrink F. Longitudinal assessment of the inflammatory response: The next step in personalized medicine after severe trauma. Front Med (Lausanne. 2022. 10.3389/fmed.2022.983259. 36203773 10.3389/fmed.2022.983259 PMC9531720

[3] van Wessem KJP, Hietbrink F, Leenen LPH. Attenuation of MODS-related and ARDS-related mortality makes infectious complications a remaining challenge in the severely injured. Trauma Surg Acute Care Open. 2020;5:e000398. 10.1136/tsaco-2019-000398. 32154377 10.1136/tsaco-2019-000398 PMC7046953

[4] Easton R, Balogh ZJ. Peri-operative changes in serum immune markers after trauma: a systematic review. Injury. 2014;45:934–41. 10.1016/j.injury.2013.12.002. 24388280 10.1016/j.injury.2013.12.002

[5] de Fraiture EJ, Bongers SH, Jukema BN, Koenderman L, Vrisekoop N, van Wessem KJP, et al. Visualization of the inflammatory response to injury by neutrophil phenotype categories: Neutrophil phenotypes after trauma. Eur J Trauma Emerg Surg. 2023;49:1023–34. 10.1007/s00068-022-02134-3. 36348032 10.1007/s00068-022-02134-3 PMC10175373

[6] Hazeldine J, Hampson P, Lord JM. The impact of trauma on neutrophil function. Injury. 2014;45:1824–33. 10.1016/j.injury.2014.06.021. 25106876 10.1016/j.injury.2014.06.021

[7] Bongers SH, Chen N, van Grinsven E, van Staveren S, Hassani M, Spijkerman R, et al. Kinetics of Neutrophil Subsets in Acute, Subacute, and Chronic Inflammation. Front Immunol. 2021;12:674079. 10.3389/fimmu.2021.674079. 34248955 10.3389/fimmu.2021.674079 PMC8265311

[8] Hellebrekers P, Hesselink L, Huisman A, ten Berg M, Koenderman L, Leenen LPH, et al. Recognizing the mobilization of neutrophils with banded nuclei early after trauma. Int J Lab Hematol. 2020;42. 10.1111/ijlh.13272. 10.1111/ijlh.13272 PMC7586805 32633074

[9] Hofman M, Andruszkow H, Kobbe P, Poeze M, Hildebrand F. Incidence of post-traumatic pneumonia in poly-traumatized patients: identifying the role of traumatic brain injury and chest trauma. Eur J Trauma Emerg Surg. 2020;46:11–9. 10.1007/s00068-019-01179-1. 31270555 10.1007/s00068-019-01179-1 PMC7223163

[10] Visser T, Hietbrink F, Groeneveld KM, Koenderman L, Leenen LPH. Isolated blunt chest injury leads to transient activation of circulating neutrophils. Eur J Trauma Emerg Surg. 2011;37:177–84. 10.1007/s00068-010-0041-x. 21837259 10.1007/s00068-010-0041-x PMC3150797

[11] Groeneveld KM, Hietbrink F, Hardcastle TC, Warren BL, Koenderman L, Leenen LPH. Penetrating thorax injury leads to mild systemic activation of neutrophils without inflammatory complications. Injury. 2014;45:522–7. 10.1016/j.injury.2013.09.030. 24119496 10.1016/j.injury.2013.09.030

[12] Dutch National Trauma Registry (DNTR). 2020. Landelijk Netwerk Acute Zorg (LNAZ). https://www.lnaz.nl/trauma/landelijke-traumaregistratie. 2024.

[13] Advanced trauma life support (ATLS^®^). J Trauma Acute Care Surg. 2013;74:1363–6. 10.1097/TA.0b013e31828b82f5. 23609291 10.1097/TA.0b013e31828b82f5

[14] Baker SP, O’Neill B, Haddon W, Long WB. The injury severity score: a method for describing patients with multiple injuries and evaluating emergency care. J Trauma. 1974;14:187–96. 4814394

[15] Barnes J, Loftis KL, Jones L, Price JP, Gillich PJ, Cookman K, et al. Development of an expert derived ICD-AIS map for serious AIS3 + injury identification. Traffic Inj Prev. 2020;21:181–7. 10.1080/15389588.2020.1725494. 32141775 10.1080/15389588.2020.1725494

[16] Rau C-S, Wu S-C, Kuo P-J, Chen Y-C, Chien P-C, Hsieh H-Y, et al. Polytrauma defined by the new berlin definition: a validation test based on propensity-score matching approach. Int J Environ Res Public Health. 2017;14. 10.3390/ijerph14091045. 10.3390/ijerph14091045 PMC5615582 28891977

[17] Association for the Advancement of Automotive Medicine. Abbreviated Injury Scale (AIS). https://www.aaam.org/abbreviated-injury-scale-ais/

[18] de Fraiture EJ, Bongers SH, Jukema BN, Koenderman L, Vrisekoop N, van Wessem KJP, et al. Visualization of the inflammatory response to injury by neutrophil phenotype categories: Neutrophil phenotypes after trauma. Eur J Trauma Emerg Surg. 2023;49. 10.1007/s00068-022-02134-3. 10.1007/s00068-022-02134-3 PMC10175373 36348032

[19] Van Gassen S, Callebaut B, Van Helden MJ, Lambrecht BN, Demeester P, Dhaene T, et al. FlowSOM: Using self-organizing maps for visualization and interpretation of cytometry data. Cytometry Part A. 2015;87:636–45. 10.1002/cyto.a.22625. 10.1002/cyto.a.22625 25573116

[20] R Core Team. A language and environment for statistical computing. R Foundation for Statistical Computing. Vienna, Austria 2017. Vienna, Austria; 2017.

[21] Kim H-Y. Statistical notes for clinical researchers: post-hoc multiple comparisons. Restor Dent Endod. 2015;40:172–6. 10.5395/rde.2015.40.2.172. 25984481 10.5395/rde.2015.40.2.172 PMC4432262

[22] Hosmer DW, Lemeshow Stanley, Sturdivant RX. Applied logistic regression. Wiley; 2013.

[23] Gennarelli TA, Wodzin Professor E. ABBREVIATED INJURY SCALE © 2005 UPDATE 2008 Editors. 2005.

[24] Vécsei V, Arbes S, Aldrian S, Nau T. Chest Injuries in Polytrauma. Eur J Trauma. 2005;31:239–43. 10.1007/s00068-005-2033-9.

[25] Grubmüller M, Kerschbaum M, Diepold E, Angerpointner K, Nerlich M, Ernstberger A. Severe thoracic trauma – still an independent predictor for death in multiple injured patients? Scand J Trauma Resusc Emerg Med. 2018;26:6. 10.1186/s13049-017-0469-7. 29310701 10.1186/s13049-017-0469-7 PMC5759165

[26] Saiz AM, Rahmati M, Gresham RCH, Baldini TD, Burgan J, Lee MA, et al. Polytrauma impairs fracture healing accompanied by increased persistence of innate inflammatory stimuli and reduced adaptive response. J Orthop Res. 2025;43:603–16. 10.1002/jor.26015. 39550711 10.1002/jor.26015 PMC11806648

[27] Störmann P, Wagner N, Köhler K, Auner B, Simon T-P, Pfeifer R, et al. Monotrauma is associated with enhanced remote inflammatory response and organ damage, while polytrauma intensifies both in porcine trauma model. Eur J Trauma Emerg Surg. 2020;46:31–42. 10.1007/s00068-019-01098-1. 30864051 10.1007/s00068-019-01098-1

[28] Bayer J, Lefering R, Reinhardt S, Kühle J, Zwingmann J, Südkamp NP, et al. Thoracic trauma severity contributes to differences in intensive care therapy and mortality of severely injured patients: analysis based on the TraumaRegister DGU^®^. World J Emerg Surg. 2017;12:43. 10.1186/s13017-017-0154-1. 28878814 10.1186/s13017-017-0154-1 PMC5581478

[29] Weckbach S, Hohmann C, Braumueller S, Denk S, Klohs B, Stahel PF, et al. Inflammatory and apoptotic alterations in serum and injured tissue after experimental polytrauma in mice: distinct early response compared with single trauma or double-hit injury. J Trauma Acute Care Surg. 2013;74:489–98. 10.1097/TA.0b013e31827d5f1b. 23354243 10.1097/TA.0b013e31827d5f1b

[30] Recknagel S, Bindl R, Kurz J, Wehner T, Ehrnthaller C, Knöferl MW, et al. Experimental blunt chest trauma impairs fracture healing in rats. J Orthop Res. 2011;29:734–9. 10.1002/jor.21299. 21437953 10.1002/jor.21299

[31] Recknagel S, Bindl R, Brochhausen C, Göckelmann M, Wehner T, Schoengraf P, et al. Systemic inflammation induced by a thoracic trauma alters the cellular composition of the early fracture callus. J Trauma Acute Care Surg. 2013;74:531–7. 10.1097/TA.0b013e318278956d. 23354247 10.1097/TA.0b013e318278956d

[32] Pape HC, Andruszkow H, Pfeifer R, Hildebrand F, Barkatali BM. Options and hazards of the early appropriate care protocol for trauma patients with major fractures: Towards safe definitive surgery. Injury. 2016;47:787–91. 10.1016/j.injury.2016.03.020. 27090109 10.1016/j.injury.2016.03.020

[33] Pape H-C, Giannoudis PV, Krettek C, Trentz O. Timing of fixation of major fractures in blunt polytrauma: role of conventional indicators in clinical decision making. J Orthop Trauma. 2005;19:551–62. 10.1097/01.bot.0000161712.87129.80. 16118563 10.1097/01.bot.0000161712.87129.80

[34] Anders H-J, Schaefer L. Beyond tissue injury-damage-associated molecular patterns, toll-like receptors, and inflammasomes also drive regeneration and fibrosis. J Am Soc Nephrol. 2014;25:1387–400. 10.1681/ASN.2014010117. 24762401 10.1681/ASN.2014010117 PMC4073442

[35] Eppensteiner J, Kwun J, Scheuermann U, Barbas A, Limkakeng AT, Kuchibhatla M, et al. Damage- and pathogen-associated molecular patterns play differential roles in late mortality after critical illness. JCI Insight. 2019;4. 10.1172/jci.insight.127925. 10.1172/jci.insight.127925 PMC6777836 31434802

[36] Lord JM, Midwinter MJ, Chen Y-F, Belli A, Brohi K, Kovacs EJ, et al. The systemic immune response to trauma: an overview of pathophysiology and treatment. Lancet. 2014;384:1455–65. 10.1016/S0140-6736(14)60687-5. 25390327 10.1016/S0140-6736(14)60687-5 PMC4729362

[37] Pape H-C, Moore EE, McKinley T, Sauaia A. Pathophysiology in patients with polytrauma. Injury. 2022;53:2400–12. 10.1016/j.injury.2022.04.009. 35577600 10.1016/j.injury.2022.04.009

[38] Vourc’h M, Roquilly A, Asehnoune K. Trauma-Induced Damage-Associated Molecular Patterns-Mediated Remote Organ Injury and Immunosuppression in the Acutely Ill Patient. Front Immunol. 2018;9:1330. 10.3389/fimmu.2018.01330. 29963048 10.3389/fimmu.2018.01330 PMC6013556

[39] Zhang Q, Raoof M, Chen Y, Sumi Y, Sursal T, Junger W, et al. Circulating mitochondrial DAMPs cause inflammatory responses to injury. Nature. 2010;464:104–7. 10.1038/nature08780. 20203610 10.1038/nature08780 PMC2843437

[40] Hatton IA, Galbraith ED, Merleau NSC, Miettinen TP, Smith BM, Shander JA. The human cell count and size distribution. Proc Natl Acad Sci. 2023;120. 10.1073/pnas.2303077120 10.1073/pnas.2303077120 PMC10523466 37722043

[41] Scozzi D, Liao F, Krupnick AS, Kreisel D, Gelman AE. The role of neutrophil extracellular traps in acute lung injury. Front Immunol. 2022;13. 10.3389/fimmu.2022.953195. 10.3389/fimmu.2022.953195 PMC9374003 35967320

[42] Doerschuk CM. Mechanisms of leukocyte sequestration in inflamed lungs. Microcirculation. 2001;8:71–88. 10.1111/j.1549-8719.2001.tb00159. 11379793

[43] Doerschuk CM, Beyers N, Coxson HO, Wiggs B, Hogg JC. Comparison of neutrophil and capillary diameters and their relation to neutrophil sequestration in the lung. J Appl Physiol (1985). 1993;74:3040–5. 10.1152/jappl.1993.74.6.3040. 8366005 10.1152/jappl.1993.74.6.3040

[44] Park I, Kim M, Choe K, Song E, Seo H, Hwang Y, et al. Neutrophils disturb pulmonary microcirculation in sepsis-induced acute lung injury. Eur Respir J. 2019;53. 10.1183/13993003.00786-2018. 10.1183/13993003.00786-2018 PMC6437604 30635296

[45] Spreadborough PJ, Strong AL, Mares J, Levi B, Davis TA. Tourniquet use following blast-associated complex lower limb injury and traumatic amputation promotes end organ dysfunction and amplified heterotopic ossification formation. J Orthop Surg Res. 2022;17:422. 10.1186/s13018-022-03321-z. 36123728 10.1186/s13018-022-03321-z PMC9484189

[46] van Eerten FJC, de Fraiture EJ, Duebel LV, Vrisekoop N, van Wessem KJP, Koenderman L, et al. Limited impact of traumatic brain injury on the post-traumatic inflammatory cellular response. Eur J Trauma Emerg Surg. 2024;50:3049–58. 10.1007/s00068-024-02574-z. 38980396 10.1007/s00068-024-02574-z PMC11666626

[47] Leliefeld PHC, Pillay J, Vrisekoop N, Heeres M, Tak T, Kox M, et al. Differential antibacterial control by neutrophil subsets. Blood Adv. 2018;2:1344–55. 10.1182/bloodadvances.2017015578. 29895625 10.1182/bloodadvances.2017015578 PMC5998927

[48] Hesselink L, Spijkerman R, de Fraiture E, Bongers S, Van Wessem KJP, Vrisekoop N, et al. New automated analysis to monitor neutrophil function point-of-care in the intensive care unit after trauma. Intensive Care Med Exp. 2020;8:12. 10.1186/s40635-020-0299-1. 32172430 10.1186/s40635-020-0299-1 PMC7072076

[49] Pillay J, Kamp VM, van Hoffen E, Visser T, Tak T, Lammers J-W, et al. A subset of neutrophils in human systemic inflammation inhibits T cell responses through Mac-1. J Clin Invest. 2012;122:327–36. 10.1172/JCI57990. 22156198 10.1172/JCI57990 PMC3248287

[50] Tak T, Wijten P, Heeres M, Pickkers P, Scholten A, Heck AJR, et al. Human CD62Ldim neutrophils identified as a separate subset by proteome profiling and in vivo pulse-chase labeling. Blood. 2017;129:3476–85. 10.1182/blood-2016-07-727669. 28515092 10.1182/blood-2016-07-727669

[51] Harwood PJ, Giannoudis PV, van Griensven M, Krettek C, Pape H-C. Alterations in the systemic inflammatory response after early total care and damage control procedures for femoral shaft fracture in severely injured patients. J Trauma. 2005;58:446–52. 10.1097/01.ta.0000153942.28015.77. discussion 452-4. 15761335 10.1097/01.ta.0000153942.28015.77

[52] van Wessem KJP, Leenen LPH, Hietbrink F. Physiology dictated treatment after severe trauma: timing is everything. Eur J Trauma Emerg Surg. 2022;48:3969–79. 10.1007/s00068-022-01916-z. 35218406 10.1007/s00068-022-01916-z PMC9532323

[53] de Fraiture EJ, Vrisekoop N, Leenen LPH, van Wessem KJP, Koenderman L, Hietbrink F. Longitudinal assessment of the inflammatory response: The next step in personalized medicine after severe trauma. Front Med (Lausanne). 2022;9:983259. 10.3389/fmed.2022.983259. 36203773 10.3389/fmed.2022.983259 PMC9531720

[54] de Fraiture EJ, Reniers T, Vreeman NEW, Rettig TCD, van Santvoort HC, Bikker A, et al. Neutrophil phenotypes quantify tissue damage caused by major surgery. Front Surg. 2025;12:1494831. 10.3389/fsurg.2025.1494831. 40124527 10.3389/fsurg.2025.1494831 PMC11925952

[55] Pfeifer R, Klingebiel FK-L, Balogh ZJ, Beeres FJP, Coimbra R, Fang C, et al. Early major fracture care in polytrauma—priorities in the context of concomitant injuries: A Delphi consensus process and systematic review. J Trauma Acute Care Surg. 2024;97:639–50. 10.1097/TA.0000000000004428. 39085995 10.1097/TA.0000000000004428 PMC11446538

[56] Moore TA, Simske NM, Vallier HA. Fracture fixation in the polytrauma patient: Markers that matter. Injury. 2020;51(Suppl 2):S10–4. 10.1016/j.injury.2019.12.024. 31879174 10.1016/j.injury.2019.12.024

[57] Timm A, Maegele M, Lefering R, Wendt K, Wyen H, TraumaRegister, DGU(^®^). Pre-hospital rescue times and actions in severe trauma. A comparison between two trauma systems: Germany and the Netherlands. Injury. 2014;45(Suppl 3):S43–52. 10.1016/j.injury.2014.08.017. 25284234 10.1016/j.injury.2014.08.017

[58] Landelijk Netwerk Acute Zorg (LNAZ). Landelijke Traumaregistratie 2018–2022: Landelijk rapportage 2023. https://www.lnaz.nl/cms/files/231101_rapport_landelijke_traumaregistratie_2018_-_2022_-_definitief.pdf. 2023.

[59] Teuben M, Heeres M, Blokhuis T, Hollman A, Vrisekoop N, Tan E, et al. Instant intra-operative neutropenia despite the emergence of banded (CD16dim/CD62Lbright) neutrophils in peripheral blood - An observational study during extensive trauma-surgery in pigs. Injury. 2021;52:426–33. 10.1016/j.injury.2020.11.018. 33208273 10.1016/j.injury.2020.11.018

[60] Sturms LM, Driessen MLS, van Klaveren D, Ten Duis H-J, Kommer GJ, Bloemers FW, et al. Dutch trauma system performance: Are injured patients treated at the right place? Injury. 2021;52:1688–96. 10.1016/j.injury.2021.05.015. 34045042 10.1016/j.injury.2021.05.015

