# Supplementary material for: Injury to the thorax is a dominant contributor to post-traumatic neutrophil mediated inflammatory response
Source: Eur J Trauma Emerg Surg. 2026 Jun 2;52(1):177. doi: 10.1007/s00068-026-03221-5 (PMC13230304; doi:10.1007/s00068-026-03221-5)
Supplement: Supplementary file 1 — Supplementary Material 1 [file 68_2026_3221_MOESM1_ESM.docx]

**Supplementary material**

**Table S1** Abbreviated Injury Scale codes used to identify injuries to body regions [20]

| **Body region** | **AIS codes*** | | | | | |
| --- | --- | --- | --- | --- | --- | --- |
| Thorax bone | "450203.3" | "450211.3" | "450213.4" | "450230.3" | "450266.5" | "451021.4" |
|  | "450209.3" | "450212.3" | "450214.5" | "450260.3" | "451020.4" | "451022.5" |
| Thorax internal | "419202.3" | "440216.4" | "441008.3" | "441411.3" | "441604.3" | "442212.5" |
|  | "419204.4" | "440400.5" | "441010.3" | "441412.4" | "441605.4" | "442602.3" |
|  | "419206.5" | "440604.3" | "441012.5" | "441414.3" | "441606.5" | "442604.3" |
|  | "419208.6" | "440606.3" | "441013.5" | "441420.3" | "442200.3" | "442606.3" |
|  | "440099.9" | "440606.4" | "441014.6" | "441422.3" | "442201.4" | "442608.4" |
|  | "440102.3" | "440608.4" | "441016.6" | "441424.4" | "442202.3" | "442610.5" |
|  | "440104.3" | "440610.4" | "441018.6" | "441426.5" | "442203.4" | "442612.4" |
|  | "440106.3" | "440804.3" | "441089.9" | "441430.3" | "442204.3" | "442614.4" |
|  | "440108.4" | "440805.3" | "441200.5" | "441431.3" | "442204.5" | "442616.5" |
|  | "440110.5" | "440806.3" | "441300.5" | "441432.4" | "442205.3" | "442699.3" |
|  | "440199.3" | "440807.3" | "441402.3" | "441450.4" | "442206.4" | "442999.9" |
|  | "440208.3" | "440808.4" | "441406.3" | "441451.4" | "442207.5" | "919202.3" |
|  | "440210.4" | "440809.4" | "441408.3" | "441452.5" | "442208.4" | "919204.4" |
|  | "440212.3" | "440810.5" | "441410.3" | "441499.3" | "442210.3" | "919206.5" |
|  | "440214.3" | "441006.4" | "441410.4" | "441603.3" | "442210.5" | "919208.6" |
| Abdomen | "500099.9" | "520604.3" | "521404.3" | "541024.4" | "542224.3" | "544414.3" |
|  | "500999.9" | "520606.3" | "521406.3" | "541025.3" | "542624.3" | "544415.3" |
|  | "510606.3" | "520608.4" | "521408.4" | "541028.5" | "542814.3" | "544416.4" |
|  | "510806.3" | "520698.4" | "521499.3" | "541224.3" | "542824.3" | "544424.3" |
|  | "511000.6" | "520699.3" | "521602.3" | "541226.4" | "542826.4" | "544426.4" |
|  | "515099.9" | "520802.3" | "521604.3" | "541424.3" | "542828.4" | "544824.3" |
|  | "515999.9" | "520804.3" | "521606.4" | "541426.4" | "542830.4" | "544826.3" |
|  | "516006.3" | "520806.4" | "521699.3" | "541614.3" | "542832.5" | "545024.3" |
|  | "520099.9" | "520899.3" | "540226.3" | "541624.3" | "543026.3" | "545026.3" |
|  | "520202.4" | "521006.3" | "540424.3" | "541626.4" | "543226.3" | "545028.3" |
|  | "520204.4" | "521102.3" | "540426.4" | "541628.5" | "543522.3" | "545028.4" |
|  | "520206.4" | "521104.3" | "540622.3" | "541640.4" | "543624.3" | "545224.3" |
|  | "520208.5" | "521106.3" | "540624.3" | "541814.3" | "543625.4" | "545226.4" |
|  | "520299.4" | "521108.4" | "540624.4" | "541824.3" | "543626.5" | "545228.5" |
|  | "520402.3" | "521199.3" | "540625.3" | "541826.4" | "543800.3" | "545230.3" |
|  | "520404.3" | "521202.3" | "540626.4" | "541828.5" | "544214.3" | "545240.3" |
|  | "520406.4" | "521204.3" | "540640.3" | "541830.6" | "544224.3" | "545426.3" |
|  | "520408.5" | "521206.4" | "540824.3" | "541840.4" | "544226.4" | "545626.3" |
|  | "520499.3" | "521299.3" | "540826.4" | "542024.3" | "544228.5" |  |
|  | "520602.3" | "521402.3" | "541023.3" | "542026.4" | "544240.3" |  |
| Lower extremity** | "800099.9" | "813002.4" | "820406.3" | "851810.3" | "853261.3" | "854162.3" |
|  | "800999.9" | "813003.3" | "820608.3" | "851812.3" | "853262.3" | "854172.3" |
|  | "810606.3" | "813004.3" | "820806.3" | "851814.3" | "853271.3" | "854222.3" |
|  | "810806.3" | "814002.3" | "821008.3" | "851822.3" | "853272.3" | "854252.3" |
|  | "811000.3" | "814006.3" | "821206.3" | "853000.3" | "853331.3" | "854262.3" |
|  | "811001.4" | "815099.9" | "830099.9" | "853001.3" | "853332.3" | "854272.3" |
|  | "811002.3" | "815999.9" | "830404.3" | "853111.3" | "853351.3" | "854332.3" |
|  | "811002.4" | "816006.3" | "830406.3" | "853112.3" | "853352.3" | "854352.3" |
|  | "811003.3" | "816013.3" | "830408.3" | "853151.3" | "853361.3" | "854362.3" |
|  | "811004.4" | "816017.3" | "840099.9" | "853152.3" | "853362.3" | "854372.3" |
|  | "811010.5" | "820099.9" | "850099.9" | "853161.3" | "853371.3" | "854456.3" |
|  | "811012.5" | "820202.3" | "851614.3" | "853162.3" | "853372.3" | "854464.3" |
|  | "812003.3" | "820204.3" | "851800.3" | "853171.3" | "853405.3" | "854466.3" |
|  | "812006.3" | "820206.3" | "851801.3" | "853172.3" | "853408.3" | "870099.9" |
|  | "812009.3" | "820208.4" | "851804.3" | "853221.3" | "853422.3" |  |
|  | "813000.3" | "820299.3" | "851808.3" | "853222.3" | "854112.3" |  |
|  | "813001.4" | "853251.3" | "854001.3" | "853252.3" | "854152.3" |  |
|  |  |  |  |  |  |  |
| *Version 2005 for >2014 and 1998 for 2013. Only AIS 3 or higher. **Pelvis excluded | | | | | | |

| **Table S2** Post-hoc analyses with Bonferonni correction for baseline characteristics and outcomes per neutrophil phenotype category |
| --- |
| \| **Age** Post-hoc Mann-Whitney-Wilcoxon with Bonferonni correction \| \| \| \| \| \| \| \| --- \| --- \| --- \| --- \| --- \| --- \| --- \| \|  \| **0** \| **1** \| **2** \| **3** \| **4** \| **5** \| \| **1** \| 1.00 \| - \| - \| - \| - \| - \| \| **2** \| 0.04 \| 0.03 \| - \| - \| - \| - \| \| **3** \| 0.02 \| 1.00 \| <0.001 \| - \| - \| - \| \| **4** \| 0.02 \| 0.50 \| <0.001 \| 1.00 \| - \| - \| \| **5** \| 1.00 \| 1.00 \| 0.42 \| 1.00 \| 1.00 \| - \| \| **6** \| 1.00 \| 1.00 \| 1.00 \| 1.00 \| 1.00 \| 1.00 \| \|  \|  \|  \|  \|  \|  \|  \| \| **Hemodynamic unstable** Post-hoc pairwise proportions with Bonferonni correction \| \| \| \| \| \| \| \|  \| **0** \| **1** \| **2** \| **3** \| **4** \| **5** \| \| **1** \| 1.00 \| - \| - \| - \| - \| - \| \| **2** \| 1.00 \| 1.00 \| - \| - \| - \| - \| \| **3** \| 1.00 \| 1.00 \| 1.00 \| - \| - \| - \| \| **4** \| 0.20 \| 1.00 \| 1.00 \| 1.00 \| - \| - \| \| **5** \| <0.001 \| 0.08 \| 1.00 \| 0.01 \| 0.76 \| - \| \| **6** \| <0.001 \| <0.001 \| 10.17 \| <0.001 \| 0.01 \| 1.00 \| \|  \|  \|  \|  \|  \|  \|  \| \| **Injury Severity Score** Post-hoc Mann-Whitney-Wilcoxon with Bonferonni correction \| \| \| \| \| \| \| \|  \| **0** \| **1** \| **2** \| **3** \| **4** \| **5** \| \| **1** \| 1.00 \| - \| - \| - \| - \| - \| \| **2** \| 1.00 \| 1.00 \| - \| - \| - \| - \| \| **3** \| <0.001 \| 0.4 \| 1.00 \| - \| - \| - \| \| **4** \| <0.001 \| <0.001 \| 0.3 \| <0.001 \| - \| - \| \| **5** \| <0.001 \| <0.001 \| <0.001 \| <0.001 \| 0.003 \| - \| \| **6** \| <0.001 \| <0.001 \| <0.001 \| <0.001 \| 0.001 \| 0.71 \| \|  \|  \|  \|  \|  \|  \|  \| \| **Glasgow coma scale <8** Post-hoc pairwise proportions with Bonferonni correction \| \| \| \| \| \| \| \|  \| **0** \| **1** \| **2** \| **3** \| **4** \| **5** \| \| **1** \| 1.00 \| - \| - \| - \| - \| - \| \| **2** \| 1.00 \| 1.00 \| - \| - \| - \| - \| \| **3** \| 1.00 \| 1.00 \| 1.00 \| - \| - \| - \| \| **4** \| 0.17 \| 1.00 \| 1.00 \| 0.56 \| - \| - \| \| **5** \| 0.24 \| 1.00 \| 1.00 \| 0.68 \| 1.00 \| - \| \| **6** \| <0.001 \| <0.001 \| <0.001 \| <0.001 \| <0.001 \| 0.08 \| \|  \|  \|  \|  \|  \|  \|  \| \| **%Banded neutrophils** Post-hoc Mann-Whitney-Wilcoxon with Bonferonni correction \| \| \| \| \| \| \| \|  \| **0** \| **1** \| **2** \| **3** \| **4** \| **5** \| \| **1** \| <0.001 \| - \| - \| - \| - \| - \| \| **2** \| <0.001 \| <0.001 \| - \| - \| - \| - \| \| **3** \| <0.001 \| 1.00 \| <0.001 \| - \| - \| - \| \| **4** \| <0.001 \| <0.001 \| 1.00 \| <0.001 \| - \| - \| \| **5** \| <0.001 \| <0.001 \| 0.003 \| <0.001 \| <.0001 \| - \| \| **6** \| 0.004 \| 1.00 \| 1.00 \| 1.00 \| 1.00 \| 1.00 \| \|  \|  \|  \|  \|  \|  \|  \| \| **Thorax injury** Post-hoc pairwise proportions with Bonferonni correction \| \| \| \| \| \| \| \|  \| **0** \| **1** \| **2** \| **3** \| **4** \| **5** \| \| **1** \| 1.00 \| - \| - \| - \| - \| - \| \| **2** \| 1.00 \| 1.00 \| - \| - \| - \| - \| \| **3** \| 0.03 \| 1.00 \| 1.00 \| - \| - \| - \| \| **4** \| <0.001 \| 0.02 \| 1.00 \| 0.01 \| - \| - \| \| **5** \| <0.001 \| 0.004 \| 0.50 \| 0.01 \| 1.00 \| - \| \| **6** \| 0.003 \| 0.10 \| 0.99 \| 0.32 \| 1.00 \| 1.00 \|  \| **Thorax bone fracture** Post-hoc pairwise proportions with Bonferonni correction \| \| \| \| \| \| \| \| --- \| --- \| --- \| --- \| --- \| --- \| --- \| \|  \| **0** \| **1** \| **2** \| **3** \| **4** \| **5** \| \| **1** \| 1.00 \| - \| - \| - \| - \| - \| \| **2** \| 1.00 \| 1.00 \| - \| - \| - \| - \| \| **3** \| 0.01 \| 1.00 \| 1.00 \| - \| - \| - \| \| **4** \| <0.001 \| 0.01 \| 1.00 \| 0.03 \| - \| - \| \| **5** \| <0.001 \| 0.09 \| 1.00 \| 1.00 \| 1.00 \| - \| \| **6** \| 0.11 \| 0.7 \| 1.00 \| 1.00 \| 1.00 \| 1.00 \| \|  \|  \|  \|  \|  \|  \|  \| \| **Thorax internal organs** Post-hoc pairwise proportions with Bonferonni correction \| \| \| \| \| \| \| \|  \| **0** \| **1** \| **2** \| **3** \| **4** \| **5** \| \| **1** \| 1.00 \| - \| - \| - \| - \| - \| \| **2** \| 1.00 \| 1.00 \| - \| - \| - \| - \| \| **3** \| 1.00 \| 1.00 \| 1.00 \| - \| - \| - \| \| **4** \| 0.02 \| 1.00 \| 1.00 \| 1.00 \| - \| - \| \| **5** \| <0.001 \| <0.001 \| 0.15 \| <0.001 \| <0.001 \| - \| \| **6** \| <0.001 \| 0.01 \| 0.58 \| <0.001 \| 0.13 \| 1.00 \| \|  \|  \|  \|  \|  \|  \|  \| \| **Abdominal injury** Post-hoc pairwise proportions with Bonferonni correction \| \| \| \| \| \| \| \|  \| **0** \| **1** \| **2** \| **3** \| **4** \| **5** \| \| **1** \| 1.00 \| - \| - \| - \| - \| - \| \| **2** \| 1.00 \| 1.00 \| - \| - \| - \| - \| \| **3** \| 1.00 \| 1.00 \| 1.00 \| - \| - \| - \| \| **4** \| 0.37 \| 0.52 \| 1.00 \| 1.00 \| - \| - \| \| **5** \| <0.001 \| <0.001 \| 1.00 \| <0.001 \| 0.07 \| - \| \| **6** \| 1.00 \| 1.00 \| 1.00 \| 1.00 \| 1.00 \| 1.00 \| \|  \|  \|  \|  \|  \|  \|  \| \| **Abdomin solid organ injury** Post-hoc pairwise proportions with Bonferonni correction \| \| \| \| \| \| \| \|  \| **0** \| **1** \| **2** \| **3** \| **4** \| **5** \| \| **1** \| 1.00 \| - \| - \| - \| - \| - \| \| **2** \| 1.00 \| 1.00 \| - \| - \| - \| - \| \| **3** \| 1.00 \| 1.00 \| 1.00 \| - \| - \| - \| \| **4** \| 1.00 \| 1.00 \| 1.00 \| 1.00 \| - \| - \| \| **5** \| <0.001 \| 0.004 \| 1.00 \| 0.001 \| 0.18 \| - \| \| **6** \| 1.00 \| - \| 1.00 \| 1.00 \| 1.00 \| 1.00 \| \|  \|  \|  \|  \|  \|  \|  \| \| **Spine injury** Post-hoc pairwise proportions with Bonferonni correction \| \| \| \| \| \| \| \|  \| **0** \| **1** \| **2** \| **3** \| **4** \| **5** \| \| **1** \| 1.00 \| - \| - \| - \| - \| - \| \| **2** \| 0.94 \| 1.00 \| - \| - \| - \| - \| \| **3** \| 0.01 \| 1.00 \| 1.00 \| - \| - \| - \| \| **4** \| <0.001 \| 1.00 \| 1.00 \| 1.00 \| - \| - \| \| **5** \| 0.12 \| 1.00 \| 1.00 \| 1.00 \| 1.00 \| - \| \| **6** \| 0.70 \| 1.00 \| 1.00 \| 1.00 \| 1.00 \| 1.00 \| \|  \|  \|  \|  \|  \|  \|  \| \| **Lower extremity injury** Post-hoc pairwise proportions with Bonferonni correction \| \| \| \| \| \| \| \|  \| **0** \| **1** \| **2** \| **3** \| **4** \| **5** \| \| **1** \| 1.00 \| - \| - \| - \| - \| - \| \| **2** \| 1.00 \| 1.00 \| - \| - \| - \| - \| \| **3** \| 1.00 \| 1.00 \| 1.00 \| - \| - \| - \| \| **4** \| 1.00 \| 1.00 \| 1.00 \| 1.00 \| - \| - \| \| **5** \| 0.02 \| 0.02 \| 1.00 \| 0.004 \| 0.11 \| - \| \| **6** \| 1.00 \| 1.00 \| 1.00 \| 1.00 \| 1.00 \| 1.00 \|  \| **Lower extremity bone injury** Post-hoc pairwise proportions with Bonferonni correction \| \| \| \| \| \| \| \| --- \| --- \| --- \| --- \| --- \| --- \| --- \| \|  \| **0** \| **1** \| **2** \| **3** \| **4** \| **5** \| \| **1** \| 1.00 \| - \| - \| - \| - \| - \| \| **2** \| 1.00 \| 1.00 \| - \| - \| - \| - \| \| **3** \| 1.00 \| 1.00 \| 1.00 \| - \| - \| - \| \| **4** \| 1.00 \| 1.00 \| 1.00 \| 1.00 \| - \| - \| \| **5** \| 0.02 \| 0.07 \| 1 \| 0.01 \| 0.12 \| - \| \| **6** \| 1.00 \| 1.00 \| 1.00 \| 1.00 \| 1.00 \| 1.00 \| \|  \|  \|  \|  \|  \|  \|  \| \| **Pelvis injury** Post-hoc pairwise proportions with Bonferonni correction \| \| \| \| \| \|  \| \|  \| **0** \| **1** \| **2** \| **3** \| **4** \| **5** \| \| **1** \| - \| - \| - \| - \| - \| - \| \| **2** \| 1.00 \| 1.00 \| - \| - \| - \| - \| \| **3** \| 0.27 \| 1.00 \| 1.00 \| - \| - \| - \| \| **4** \| 0.001 \| 0.29 \| 1.00 \| 0.63 \| - \| - \| \| **5** \| <0.001 \| 0.004 \| 1.00 \| 0.004 \| 1.00 \| - \| \| **6** \| 0.64 \| 1.00 \| 1.00 \| 1.00 \| 1.00 \| 1.00 \| \|  \|  \|  \|  \|  \|  \|  \| \| **Length of hospital stay** Post-hoc Mann-Whitney-Wilcoxon with Bonferonni correction \| \| \| \| \| \| \| \|  \| **0** \| **1** \| **2** \| **3** \| **4** \| **5** \| \| **1** \| 1.00 \| - \| - \| - \| - \| - \| \| **2** \| 1.00 \| 1.00 \| - \| - \| - \| - \| \| **3** \| <0.001 \| 1.00 \| 1.00 \| - \| - \| - \| \| **4** \| <0.001 \| 0.01 \| 0.29 \| 0.01 \| - \| - \| \| **5** \| <0.001 \| 0.003 \| 0.03 \| 0.002 \| 0.50 \| - \| \| **6** \| 1.00 \| 1.00 \| 1.00 \| 1.00 \| 0.53 \| 0.21 \| \|  \|  \|  \|  \|  \|  \|  \| \| **Length of ICU stay** Post-hoc Mann-Whitney-Wilcoxon with Bonferonni correction \| \| \| \| \| \| \| \|  \| **0** \| **1** \| **2** \| **3** \| **4** \| **5** \| \| **1** \| 1.00 \| - \| - \| - \| - \| - \| \| **2** \| 1.00 \| 1.00 \| - \| - \| - \| - \| \| **3** \| 0.12 \| 1.00 \| 1.00 \| - \| - \| - \| \| **4** \| <0.001 \| 0.14 \| 1.00 \| 0.01 \| - \| - \| \| **5** \| <0.001 \| 0.003 \| 0.09 \| <0.001 \| 0.69 \| - \| \| **6** \| <0.001 \| <0.001 \| <0.001 \| <0.001 \| 0.02 \| 1.00 \| \|  \|  \|  \|  \|  \|  \|  \| \| **Mortality** Post-hoc pairwise proportions with Bonferonni correction \| \| \| \| \| \| \| \|  \| **0** \| **1** \| **2** \| **3** \| **4** \| **5** \| \| **1** \| 1.00 \| - \| - \| - \| - \| - \| \| **2** \| 1.00 \| 1.00 \| - \| - \| - \| - \| \| **3** \| 0.46 \| 1.00 \| 1.00 \| - \| - \| - \| \| **4** \| 0.001 \| 0.67 \| 1.00 \| 0.54 \| - \| - \| \| **5** \| 1.00 \| 1.00 \| 1.00 \| 1.00 \| 1.00 \| - \| \| **6** \| <0.001 \| <0.001 \| 0.001 \| <0.001 \| <0.001 \| 0.002 \| |

| **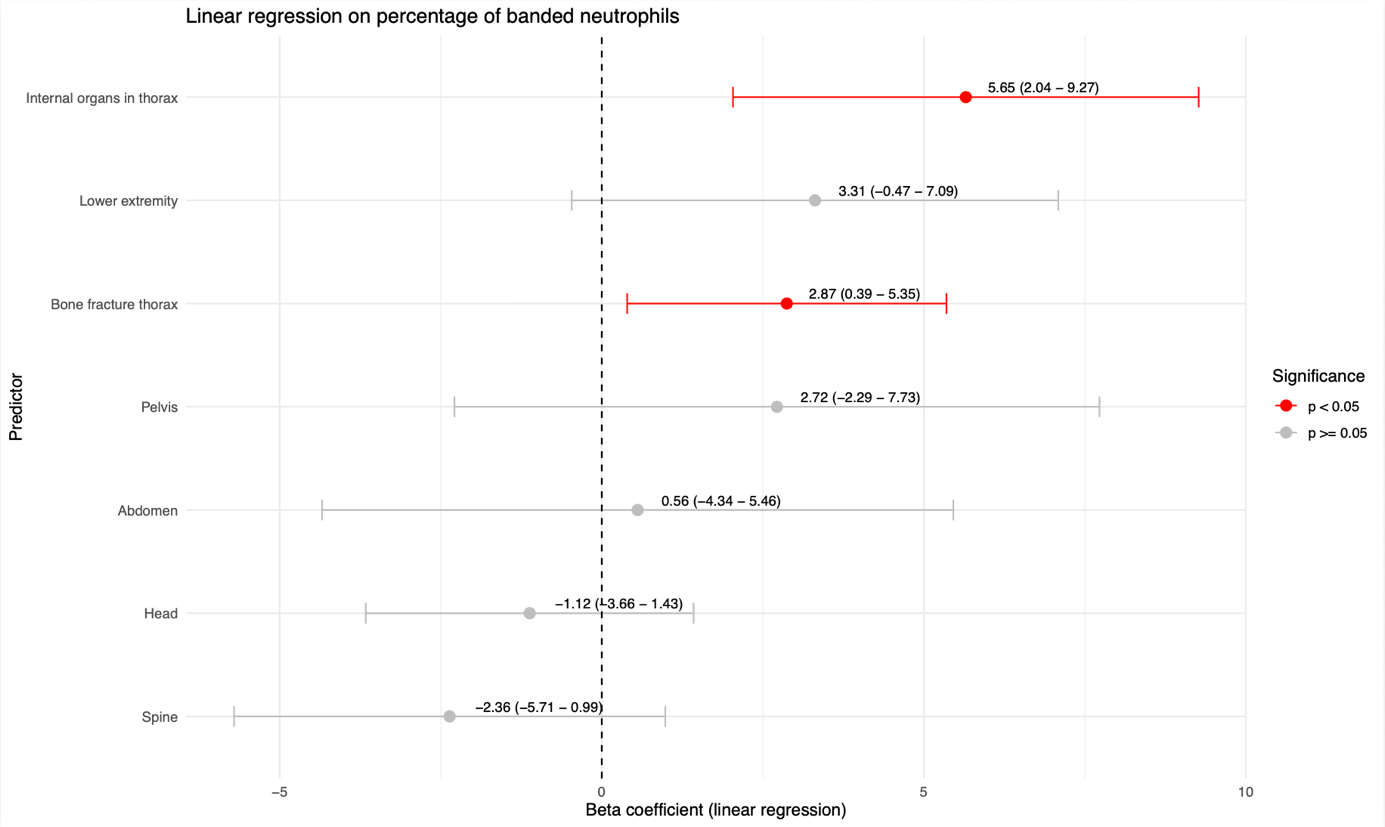** |
| --- |
| **Fig. S1** Linear univariable regression on percentage of banded neutrophils present, i.e. extensive post-traumatic inflammatory response. The predictors were analyzed together in one model, adjusting for Injury Severity Score (ISS), age and hemodynamic instability as confounders. Hemodynamic instability was defined as a systolic blood pressure <90 mmHg at emergency department arrival. |

| **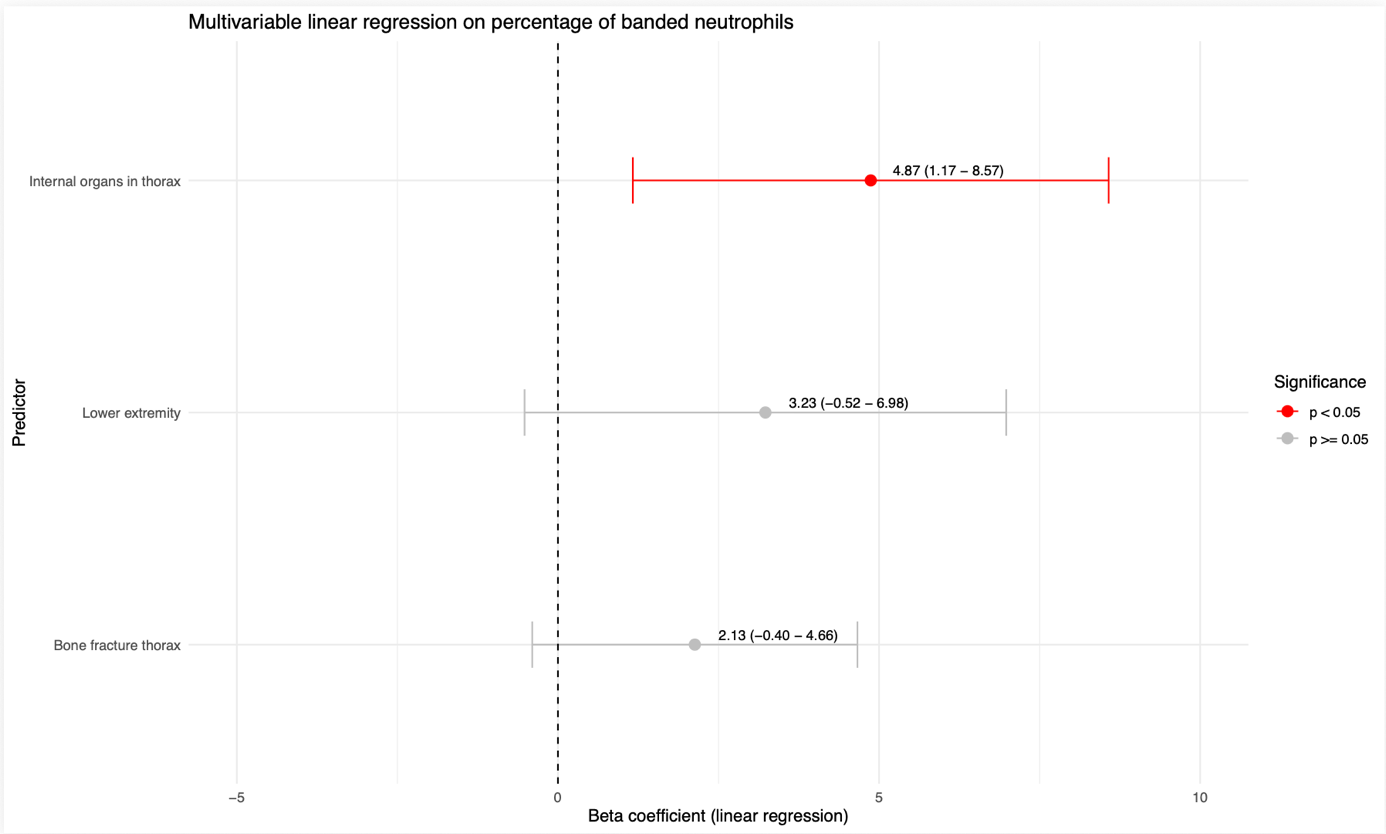** |
| --- |
| **Fig. S2** Linear multivariable regression on percentage of banded neutrophils present; i.e. extensive post-traumatic inflammatory response. The predictors were analyzed together in one model, adjusting for Injury Severity Score (ISS), age and hemodynamic instability as confounders. Hemodynamic instability was defined as a systolic blood pressure <90 mmHg at emergency department arrival. |

| 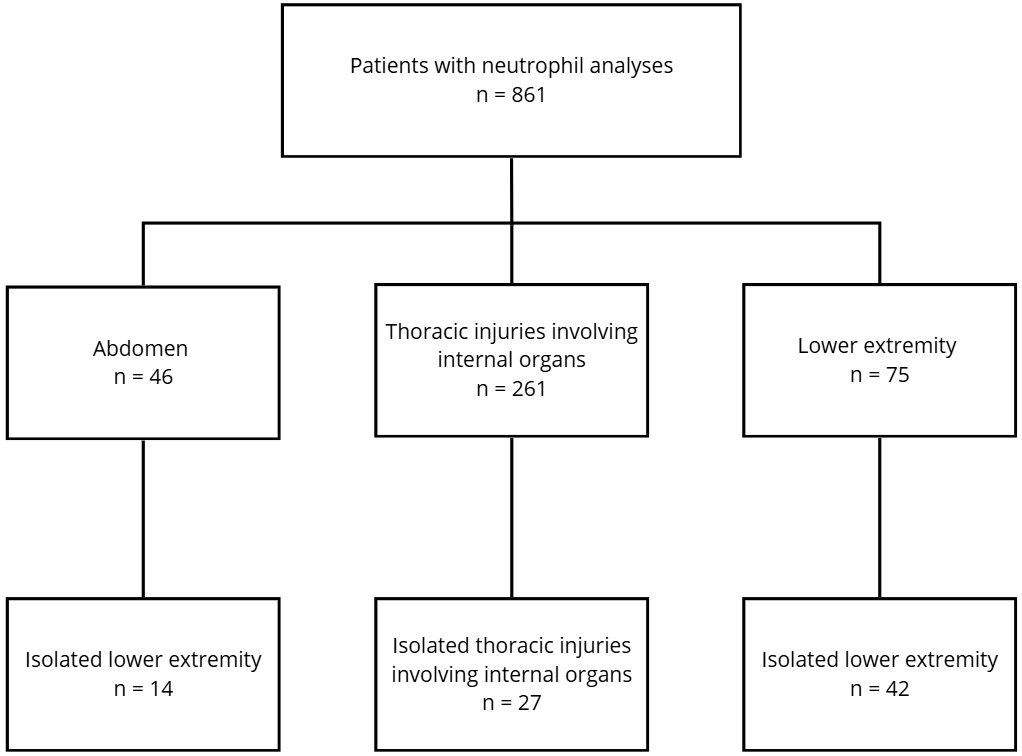 |
| --- |
| **Fig. S3** flowchart of patients included in the analysis on inflammatory response in patients who suffered from thoracic injuries compared to other injuries |
